# Effect of High‐Fidelity Simulation on Self‐Efficacy, Empathy and Satisfaction Among Undergraduate Paediatric Nursing Students: A Quasi‐Experimental Study

**DOI:** 10.1002/nop2.70993

**Published:** 2026-11-13

**Authors:** Yasmine Alabbasi, Hibah Bahri, Ahlam Almutairi, Raghad Alqahtani, Fatima Alsufayan, Ghaida Alturaysi, Renad Alshalawi, Lamya Atin, Renad Aljuhani, Savvato Karavasileiadou

**Affiliations:** ^1^ Department of Maternity and Pediatric Nursing, College of Nursing Princess Nourah bint Abdulrahman University Riyadh Saudi Arabia; ^2^ Department of Medical‐Surgical Nursing, College of Nursing Princess Nourah bint Abdulrahman University Riyadh Saudi Arabia; ^3^ College of Nursing, Princess Nourah bint Abdulrahman University Riyadh Saudi Arabia; ^4^ Department of Community and Psychiatric Mental Health Nursing College of Nursing, Princess Nourah bint Abdulrahman University Riyadh Saudi Arabia

## Abstract

**Background:**

Simulation is a critical factor in the training of nursing profession, offering a safe environment for practical learning to replicate real‐world clinical scenarios.

**Aim:**

This study aims to assess the impact of high‐fidelity simulation labs in nursing education on self‐efficacy, empathy and satisfaction among paediatric nursing students.

**Methods:**

A one group quasi‐experimental study with a pre‐test and post‐test design was conducted with 143 undergraduate nursing students, who were exposed to two simulations scenarios. A pre‐test was conducted to assess students' baseline General Self‐Efficacy Scale (GSE), Kiersma‐Chen Empathy Scale (KCES‐R) before any intervention. Student performance was then measured with a post‐test immediately after the training to measure self‐efficacy, empathy and the Student Satisfaction and Self‐Confidence in Learning (SCL).

**Results:**

The results showed a statistically significant difference in mean scores between the pre‐test and post‐test group in terms of self‐efficacy (30.22 vs. 33.50; *p* < 0.001; CI = 2.10–4.43). However, there is no significant difference between pre/post self‐empathy (KCES‐R). The Pearson correlation test showed a correlation between students' satisfaction and self‐confidence in learning, with self‐efficacy *p* < 0.05.

**Conclusions:**

The results of this study confirmed the effectiveness of high‐fidelity simulation in enhancing self‐efficacy of nursing paediatric students. However, further research is needed to understand their impact on empathy. The development of empathy may require additional structured interventions, such as guided debriefing sessions that focus specifically on the emotional and interpersonal aspects of patient care.

**Implications for the Profession and/or Patient Care:**

Self‐efficacy, satisfaction and empathy are interrelated factors that significantly impact paediatric nursing students' educational experiences and professional readiness. Enhancing these attributes through targeted educational strategies can lead to improved academic performance, better patient care and a more competent nursing workforce.

**Reporting Method:**

This study adhered to the TREND (Transparent Reporting of Evaluations with Nonrandomized Designs) guidelines for reporting non‐randomized intervention evaluations.

**Patient or Public Contribution:**

No patient or public contribution was required for this study, as the research focused on evaluating an educational intervention among university nursing student.

## 1 Introduction

High‐fidelity simulation (HFS) is a pedagogical approach that utilizes advanced computerized patient simulators to replicate real‐world clinical scenarios, allowing nursing students to practice skills without risking patient safety (Gordon et al. 2001). Simulation labs serve as an essential training method in nursing education, effectively bridging the gap between theoretical knowledge and practical application through innovative teaching strategies (Gordon et al. 2001; Koukourikos et al. 2021). Simulation‐based education in nursing programmes in Saudi Arabia has gained significant traction, particularly in enhancing clinical skills and improving educational outcomes. Nursing students generally express positive attitudes toward simulation, recognizing its importance in preparing them for real‐world practice, with studies indicating that approximately 91.3% of students appreciate its role in their education (Eltaib et al. 2024). The COVID‐19 pandemic prompted the integration of video‐based simulations, allowing for continued learning while adhering to health guidelines (Nguyen et al. 2023). Faculty readiness plays a crucial role in successful implementation, with many faculty members feeling prepared to adopt simulation into their curricula, as evidenced by a study showing significant faculty support for simulation‐based education (Almotairy et al. 2023).

Research has also shown that the use of high‐fidelity manikins in nursing education leads to improved nursing students' clinical competency, critical thinking, communication and psychomotor skills and enables independent decision‐making within a psychologically safe environment (Oermann and Gaberson 2021; Mohamed and Fashafsheh 2019; Lewis et al. 2022). Qualitative findings further confirm that these secure, simulated spaces effectively build student confidence and communication abilities (Jacobs and Venter 2017). Building on this foundation, combining simulation practicums with flipped learning significantly enhances clinical skills, self‐efficacy and overall student competency compared to conventional teaching approaches (Gu and Sok 2020).

Satisfaction, in the context of undergraduate nursing students utilizing high‐fidelity simulation, refers to the degree to which students feel fulfilled, content and positive about their learning experiences during simulated clinical scenarios (Toqan et al. 2023; Oanh et al. 2021). It includes positive learning outcomes, where students feel their clinical skills and knowledge improve, high engagement during realistic scenarios that enhance participation and emotional responses that foster confidence and reduce anxiety. Constructive feedback and reflection following simulations are crucial for identifying strengths and areas for growth, while peer interaction promotes a sense of community and teamwork. Collectively, these elements contribute to fulfilling educational experiences, influencing students' motivation and preparedness for clinical practice (Toqan et al. 2023; Oanh et al. 2021).

Higher l student drives better retention, academic performance and professional engagement in nursing education. Satisfaction also correlates with self‐efficacy; students who are satisfied with their education tend to have higher self‐efficacy beliefs, which further enhances their learning outcomes (Nguyen et al. 2026).

Research in Saudi Arabia and Jordan highlighted the positive impact of simulation labs on nursing students' satisfaction. In Saudi Arabia, students reported high levels of satisfaction and motivation from clinical simulation activities (Mujamammi et al. 2024). Similarly, Jordanian students rated their satisfaction with virtual reality simulation‐based learning above average, appreciating the teaching methods (Salameh et al. 2024). A notable correlation was found between satisfaction and self‐confidence, suggesting that increased self‐confidence in using simulations leads to greater satisfaction with the learning experience. This interconnected relationship holds true across distinct nursing specialties; whether tackling mental health scenarios or practicing airway management, high‐fidelity simulation consistently outperforms standard instruction by simultaneously driving up students' practical skills, satisfaction and self‐confidence (Jawabreh et al. 2025; Hodrob et al. 2024).

Self‐efficacy describes a person's confidence in their own capability to carry out tasks and handle particular situations effectively. In the context of nursing education, self‐efficacy is crucial as it influences students' motivation, learning and performance. Research indicates that higher levels of self‐efficacy are associated with better academic outcomes and clinical performance (Albaqawi et al. 2025). Strategies to enhance self‐efficacy, such as simulation‐based learning, have been shown to improve nursing students' critical thinking skills, thereby preparing them for real‐world scenarios (Jawabreh et al. 2025; Hodrob et al. 2024; Doğan and Şendir 2022). Engaging in simulated scenarios allows students to practice and apply their knowledge in a controlled environment, thereby increasing their confidence in their skills. A quasi‐experimental study showed that learners in both the high‐fidelity and virtual simulation groups experienced a significant increase in self‐efficacy after the training (Albaqawi et al. 2025; Guerrero et al. 2024). This underscores the importance of simulation as a vital educational tool in nursing curricula for fostering self‐efficacy, regardless of its placement within the programme.

Empathy in professional care is best understood not as a solitary trait, but as a dynamic, interactive relationship built through the shared experiences of both individuals (Doğan and Şendir 2022; Van Dijke et al. 2020). Developing empathy is crucial for delivering high‐quality patient care, necessitating that nursing students acquire relevant cognitive, emotional and practical skills. A study has shown that empathy is associated with enhanced emotional support and caring behaviours (Van Dijke et al. 2020; Atta et al. 2024). Furthermore, educational interventions that focus on enhancing empathy can lead to better clinical outcomes and a more resilient nursing workforce (Atta et al. 2024; Ayed et al. 2021; Ayed et al. 2021; Deligianni et al. 2017; Moudatsou et al. 2020). Recent meta‐analytic evidence indicates that simulation‐based education can improve nursing students' empathy, with newer studies showing even stronger positive results (Cho and Kim 2024b).

Self‐efficacy, satisfaction and empathy are interrelated factors that significantly impact nursing students' educational experiences and professional readiness. Enhancing these attributes through targeted educational strategies can lead to improved academic performance, better patient care and a more competent nursing workforce.

Nursing schools are facing challenges related to expanding enrollment, retention and preparing students for the Saudi Nursing Licensure Examination (SNLE) amid a growing nursing shortage. To address these issues, schools are exploring ways to identify at‐risk students and implement interventions that enhance their academic success, as SNLE pass rates are crucial for programme accreditation and professional licensure.

However, nursing students, particularly in paediatrics, often lack hands‐on practice due to various restrictions and limited opportunities in clinical settings. These challenges, along with patient safety protocols, can hinder student engagement in care. Although prior educational studies show that high‐fidelity simulation helps build general technical competence, there remains limited empirical evidence regarding the immediate changes in student empathy and self‐efficacy following exposure to high‐fidelity paediatric scenarios. Further research is necessary to understand how simulation impacts paediatric nursing students' empathy, self‐efficacy and satisfaction.

This study aims to assess the impact of high‐fidelity simulation labs in nursing education on self‐efficacy, satisfaction and empathy among paediatric nursing students. The overarching objective is to improve their readiness for clinical practice and increase their chances of successfully passing the SNLE.

## 2 Materials and Methods

### 2.1 Design

A one‐group pre‐test and post‐test quasi‐experimental design was used for this study. It aimed to determine the effect of the simulation lab on self‐efficacy, empathy and satisfaction among undergraduate nursing students who enrolled in a paediatric nursing course.

### 2.2 Research Tools

Following the demographic data section, three sets of questionnaires were used to gather data related to self‐confidence, efficacy and empathy: (1) the Student Satisfaction and Self‐Confidence in Learning Questionnaire (SCL), (2) the General Self‐Efficacy Scale (GSE) and lastly, (3) the Empathy Scale of Kiersma–Chen Empathy Scale (KCES‐R).

The second section of the questionnaire is the psychological General Self‐Efficacy Scale (GSE), which was used to evaluate the learners' self‐efficacy levels in order to understand their self‐beliefs in coping with various challenging situations (Deligianni et al. 2017; Schwarzer and Jerusalém 1995). The GSE is a 10‐item test used to gauge a person's perception of their ability to deal with a variety of difficult situations in life. The General Self‐Efficaciousness Scale has a maximum score of 40 and a scoring range of 10–40. A higher score indicates more self‐efficacy. The average time it takes to complete the scale is 4 min. With Cronbach's scores ranging from 0.75 to 0.90, samples from 23 different nations have been used to illustrate the reliability of the scale.

The KCES‐R is the third section, and it is used to measure the emotional and cognitive components of empathy in nursing students. Dr. Mary Kiersma created and validated it. It is used to evaluate empathy levels. The test is divided into two sections. Section one measures the students' perception of the importance of healthcare providers being able to demonstrate empathy, and section two pretrained to the students' perception of their own ability to demonstrate empathy toward others. It has 14 elements covering numerous aspects of empathy, such as comprehending patients' feelings, acting compassionately and using active listening techniques. Using a seven‐point Likert scale. A higher score indicates more empathy. Cronbach alpha for seven factors (global views) was 0.84 and 0.77 for four factors (personal ability) (Moudatsou et al. 2020; Aronson et al. 2022). In the present study, Cronbach's alpha was 0.89 and 0.75 for the two KCES‐R subscales, respectively and 0.89 for the overall scale.

The student satisfaction and self‐confidence in learning (SCL) section is the fourth one. The scale is used to measure the students' satisfaction and self‐confidence levels after their simulation experience. It is a 13‐item, 5‐point Likert scale developed by the National League for Nursing (Cho and Kim 2024b; Franklin et al. 2014). Adapted version of the Student Satisfaction and Self‐Confidence in Learning Scale was used. Items 2 and 7 were adapted to change the focus from a medical‐surgical curriculum to a paediatric medical‐surgical. Consistent with standard administration protocols for the SCL scale, measurements were taken exclusively post‐simulation to capture students' evaluations of the specific paediatric scenario, instruction and debriefing provided. The instrument consists of two sub‐scales: the first, consisting of five items, gauges student pleasure with learning through simulation, while the second, consisting of eight items, gauges student confidence with learning through simulation. Greater satisfaction is indicated by higher scores. Scores on the pleasure sub‐scale range from 5 to 25, while scores on the self‐confidence sub‐scale range from 8 to 40. The content validity of the measure was assessed by nine clinical specialists (Jeffries and Rizzolo 2006). The sources published state that the self‐confidence and satisfaction sub‐scales have a Cronbach's alpha coefficient for satisfaction of 0.94 and confidence of 0.87, assessing optimistic self‐beliefs to cope with a variety of difficult demands in life.

### 2.3 Study Population

The study focused on undergraduate nursing students from the third year, level six, who attended an academic institution and enrolled in the paediatric nursing care course. According to the official nursing programme study plan, paediatric nursing is introduced in the third year. While third‐year participants had prior simulation experience in lower‐level courses (Adult Nursing), this course represented their first formal exposure to paediatric‐specific clinical scenarios and concepts.

### 2.4 Sample Size and Sampling Technique

The total number of students enrolled in the paediatric course was 317. The sample size was based on the change in the General Self‐Efficacy Scale (GSE) total score from pre‐ to post‐simulation as the primary outcome. For the paired pre–post design, a conservative expected paired effect size (Cohen's dz. = 0.30), a significance level of 0.05% and 80% power were used. The minimum required sample size was approximately 90 students.

Using a convenience sampling approach, students were invited to participate. A total of 148 students agreed to participate (100% response rate) and completed both the pre‐test and post‐test assessments. Five students completed an initial pilot to evaluate scenario timing and questionnaire clarity and applicability; their data were excluded from the primary analysis.

### 2.5 Data Collection

Once ethical approval was obtained, a pilot survey was administered to five nursing students to evaluate feasibility, clarity and applicability and were excluded from the final analyses. No modifications were required, as the students confirmed the clarity of the survey. The remaining 143 students completed both the pre‐test and post‐test assessments, forming the final analytic sample (*n* = 143).

Data were collected in 2024. The questionnaire was uploaded on Google Forms and distributed via the student affairs offices of the College of Nursing and Deanship of Scientific Research to students' emails. The participants completed the questionnaire within 5–10 min for the pre‐test (the self‐efficacy and empathy scales) and 7–15 min for the post‐test (self‐efficacy, empathy and Student Satisfaction and Self‐Confidence in Learning scales) during their clinical training in simulation on the paediatric nursing course. The pre‐test was completed immediately before the simulation began, and the post‐test was administered right after the session ended while students were still in the simulation lab. No intensive was provided.

### 2.6 Inclusion and Exclusion Criteria

Participants who met the following criteria were included:

- Full‐time undergraduate nursing students.
- Third‐year (Level 6) students, as the paediatric nursing course is offered to third‐year students (Level 6).
- Students enrolled in the paediatric nursing course and took the course for the first time.
- Students who had prior simulation experience

Students who did not meet the inclusion criteria were excluded.

### 2.7 Variables to Study

The variables were determined to achieve the aims of the present study, which were mainly to evaluate the effect of high‐fidelity simulation on students' self‐efficacy, empathy and satisfaction pre–post‐intervention in undergraduate paediatric nursing students at an academic institution in Saudi Arabia. The variables included the following: self‐efficacy, empathy and satisfaction.

### 2.8 Ethical Consideration

The study was conducted according to the guidelines of the Declaration of Helsinki and was approved by the Institutional Review Board of Princess Nourah bint Abdulrahman University (IRB Log Number: 24–0660). A convenient sample of eligible participants was voluntarily invited through email announcements from the Deanship of Scientific Research. Consent forms were signed by participants before the data collection, and the confidentiality and privacy of the participants in the current study were ensured, with emphasis on their right to leave the survey at any time point.

### 2.9 Data Analysis

The data were analyzed using SAS version 9.4 (SAS Institute Inc., Cary, NC, USA) (Schwarzer and Jerusalém 1995; SAS Institute Inc. 2023). Descriptive statistics were used to summarize the participants' demographic data, with frequency and percentage used for categorical variables and mean and standard deviation for continuous variables. The normality of the data was assessed using the Shapiro–Wilk test prior to conducting the paired *t*‐tests. A paired *t*‐test was used to evaluate changes from pre‐ to post‐test for the composite and subscale scores. Cohen's dz. with 95% confidence intervals was reported as the standardized effect size. Regarding missing data, all questionnaires were checked for completeness before analysis, resulting in no missing values for the study variables. The Pearson correlation test was also utilized to evaluate the correlation between continuous variables. The significance level was set at *p* ≤ 0.05.

### 2.10 Intervention Procedure

The two‐week intervention involved simulation scenarios and debriefing. Specifically, two simulation scenarios, urinary tract infection followed by an anaphylactic reaction and neonatal respiratory depression, were selected because they represent critical, high‐risk clinical events that require rapid assessment and timely intervention. These scenarios were chosen to challenge students appropriately and provide an optimal environment for evaluating changes in their confidence, empathy and clinical judgement. The objectives of the scenarios were aligned with the course learning outcomes. The International Nursing Association for Clinical Simulation and Learning (INACSL) Standards of Best Practice (simulation) were followed. The simulation was held at the simulation centre using a high‐fidelity paediatric simulator. Before the simulation, students were instructed to review resources to ensure they were familiar with the content. All the study participants had orientation sessions that introduced students to the simulation room, paediatric high‐fidelity simulator, actions, procedures, professional expectations, confidentiality and rules of the simulation centre.

The nursing instructor described simulation learning objectives and briefly reported the patient's case (Figure 1). All students attended the two intervention sessions. There was a total of six sections; each section was divided into four to five groups, and roles were assigned to students. The technician and the instructor operated the simulator from a one‐way mirror glass control room adjacent to the simulation room and spoke via a microphone at the beginning and end of the simulation. The scenario lasted for almost 20 min. A structured debriefing session was conducted in a private meeting room adjacent to the simulation room to facilitate oral reflective debriefing. Instructors provided reflective feedback to encourage students to contemplate their actions and decisions, followed by constructive feedback that offered suggestions for improvement. Adherence to the simulation protocol was assessed, including compliance with the simulation guidelines, ensuring active participation, fulfilling assigned roles and responsibilities, applying consistent decision‐making and maintaining professional behaviour.

**FIGURE 1:**
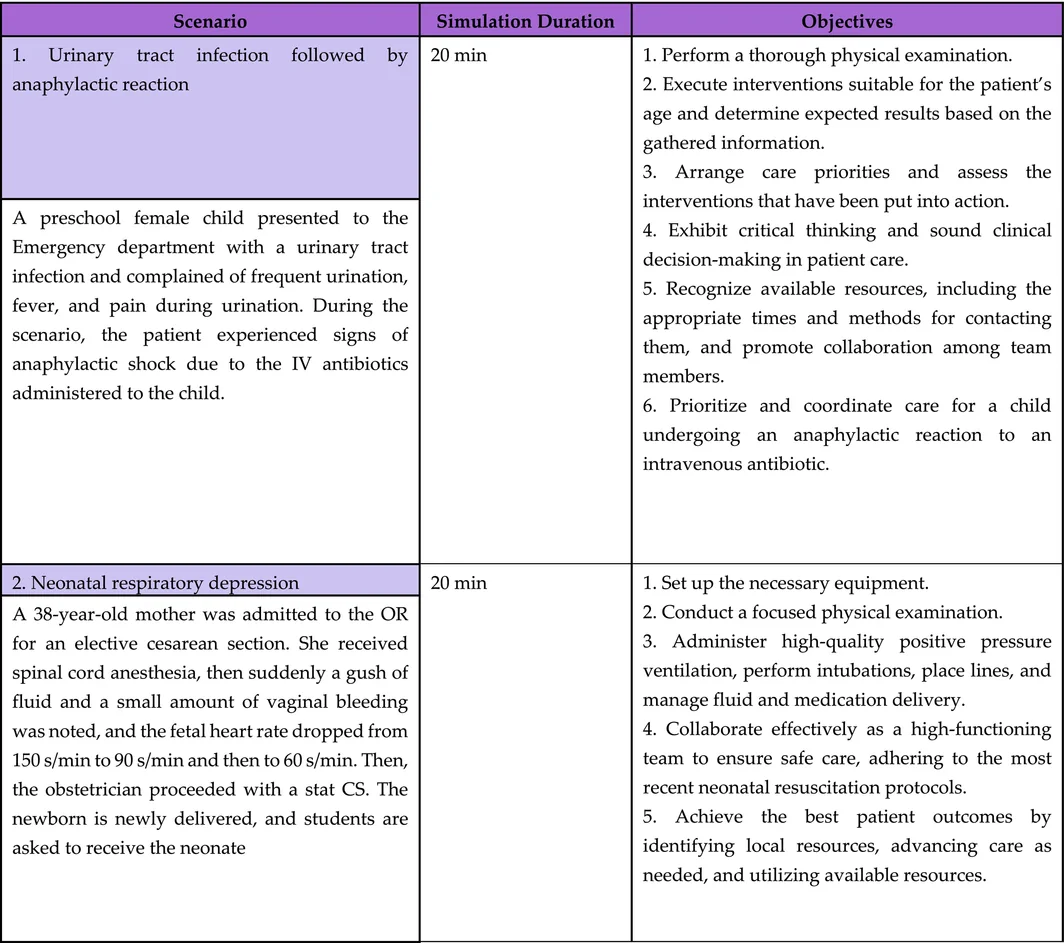
Simulation scenarios.

## 3 Results

### 3.1 Descriptive Characteristics

Of the 143 students who were enrolled in the paediatric nursing course who participated in this study, 83 (58.04%) participants were in the age group (19–20 years), 85 (59.44%) had a GPA between 4.0 and 4.9 and 143 (100%) had previous experience in simulation labs (Table 1).

**TABLE 1:** Descriptive characteristics (*n* = 143).

Characteristic	Frequency	(%)
Age		
19–20	83	58.04
21–22	56	39.16
23–24	4	2.80
GPA		
3.0–3.9	57	39.86
4.0–4.9	85	59.44
5	1	0.70
Specialty		
Nursing	143	100.00
Previous experience in simulation lab		
Yes	143	100.00
Total	143	100

### 3.2 General Self‐Efficacy Outcomes

A Paired *t*‐test was used to compare pre‐ and post‐GSE composite scores. Students responded to each item on the General Self‐Efficacy Scale (GSE) using a 4‐point Likert scale (1 = Not at all true, 2 = Hardly true, 3 = Moderately true, 4 = Exactly true). The Paired *t*‐test showed that students' overall self‐efficacy significantly increased from a mean composite score of 30.22–33.50 (*p* < 0.0001; Table 2).

**TABLE 2:** Differences between pre‐ and post‐General Self‐Efficacy Scale (GSE) scores.

GSE question	Pre‐mean	Post‐mean	Mean change	95% CI	*p*	Cohen's dz (95% CI)
1. I can always manage to solve difficult problems if I try hard enough.	3.15	3.30	—	—	—	—
2. If someone opposes me, I can find the means and ways to get what I want.	2.91	3.35	—	—	—	—
3. It is easy for me to stick to my aims and accomplish my goals.	2.99	3.34	—	—	—	—
4. I am confident that I could deal efficiently with unexpected events.	3.01	3.41	—	—	—	—
5. Thanks to my resourcefulness, I know how to handle unforeseen situations.	2.91	3.36	—	—	—	—
6. I can solve most problems if I invest the necessary effort.	3.17	3.39	—	—	—	—
7. I can remain calm when facing difficulties because I can rely on my coping abilities.	2.94	3.37	—	—	—	—
8. When I am confronted with a problem, I can usually find several solutions.	2.99	3.38	—	—	—	—
9. If I am in trouble, I can usually think of a solution.	3.15	3.32	—	—	—	—
10. I can usually handle whatever comes my way.	3.00	3.27	—	—	—	—
Total score	30.22	33.50	3.27	2.10–4.43	< 0.0001 *	0.47 (0.30–0.64)

* *p* < 0.05 was considered statistically significant.

### 3.3 Kiersma–Chen Empathy Outcomes

A Paired *t*‐test was used to compare the differences between pre‐ and post KCES‐R scores. Students responded to each item on the tool using a 7‐point Likert scale reflecting the degree of agreement. The test was divided into two sections. Section one showed a significant decrease in the overall subscale score (*p* < 0.05), whereas no significant difference was found in the overall section two score. Additionally, there was no significant difference between the total pre/post (KCES‐R) mean scores (*p* > 0.05). The pre‐test mean was (72.58) and the post‐test mean was (70.97). The study‐specific reliability was assessed using Cronbach's alpha. The coefficients were 0.89 and 0.75 for the two KCES‐R subscales, respectively, and 0.89 for the overall scale (Table 3).

**TABLE 3:** Differences between pre‐ and post‐Kiersma–Chen Empathy Scale (KCES‐R) Scores.

	Pre‐mean	Post‐mean	Mean change	95% CI	*p*	Cohen's dz (95% CI)
Section 1. The students' perception of the importance of healthcare providers being able to demonstrate empathy						
1. How necessary is it to comprehend a patient's experiences.	5.52	4.94	—	—	—	—
2. How necessary is it to be caring in order to build a strong relationship with a patient	5.63	5.29	—	—	—	—
3. How necessary is it to value a patient's point of view.	5.75	5.41	—	—	—	—
4. How necessary is it to consider a patient's feelings to provide patient‐centred care.	4.93	5.01	—	—	—	—
5 How necessary is it to be caring in order to build a strong relationship with a patient.	5.87	5.36	—	—	—	—
6. How necessary is it to identify with a patient's feelings.	5.44	5.31	—	—	—	—
7. How necessary is it to view the world from a patient's perspective.	5.37	5.21	—	—	—	—
Total Section 1 score	38.50	36.54	−1.96	−3.85 to −0.07	0.0416*	−0.17 (−0.33 to −0.001)
Section 2. The students' perception of their own ability to demonstrate empathy toward others						
8. I am able to comprehend a patient's experiences.	5.25	4.91	—	—	—	—
9. I am able to express an understanding of a patient's feelings.	5.29	5.06	—	—	—	—
10. I am able to value a patient's point of view.	5.41	5.22	—	—	—	—
11. I am able to consider feelings to provide patient‐centred care.	3.14	3.97	—	—	—	—
12. I am able to be caring in order to build a strong relationship with a Patient	5.85	5.57	—	—	—	—
13. I am able to identify with a patient's feelings.	4.08	4.51	—	—	—	—
14. I am able to view the world from a patient's perspective	5.06	5.21	—	—	—	—
Total Section 2 score	34.08	34.43	0.34	−1.32 to 2.02	0.6808	0.03 (−0.13 to 0.20)
Total KCES‐R Score	72.58	70.97	−1.61	−4.91 to 1.68	0.3365	−0.08 (−0.24 to 0.08)

* *p* < 0.05 was considered statistically significant.

### 3.4 Comparison Between Total Pre–Post Mean Change of Self‐Efficacy and Empathy Scale Scores

Students' overall self‐efficacy significantly increased from a mean composite score of 30.22–33.50 (*p* < 0.0001). However, there is no significant difference between pre and post (KCES‐R) mean scores (*p* > 0.05). The pre‐test mean was 72.58, and the post‐test mean was 70.97 (Figure 2).

**FIGURE 2:**
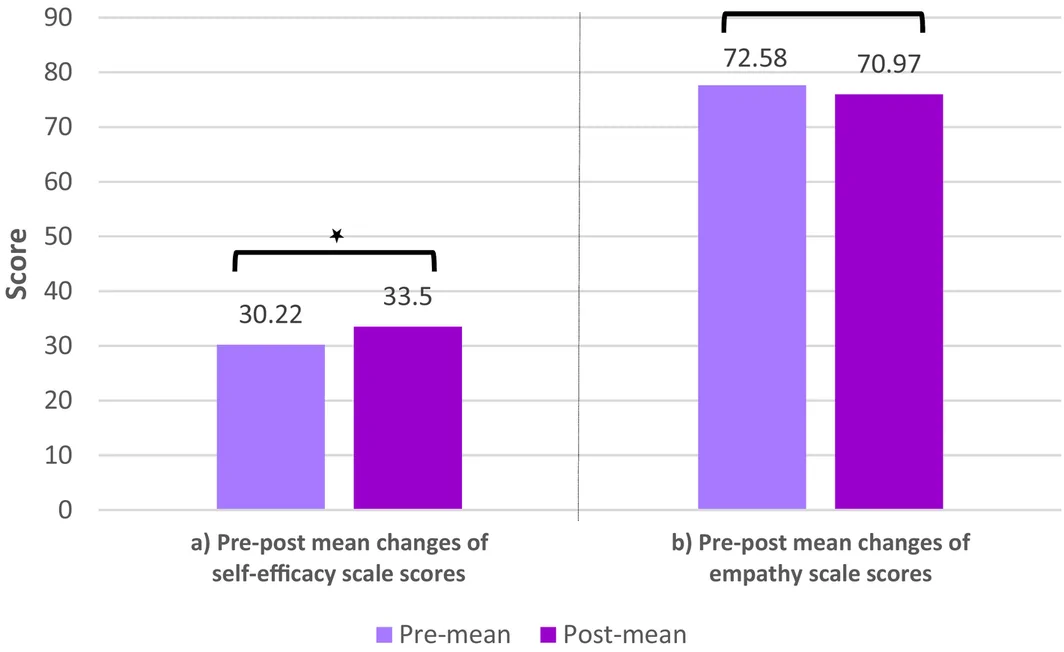
Comparison between total pre‐ and post‐mean change in Self‐Efficacy and Empathy Scale scores (a); total pre‐mean and post‐mean change of Self‐Efficacy Scale scores (b); total pre‐mean and post‐mean change of empathy scale scores. Bars represent the mean. *p < 0.001.

### 3.5 Students' Satisfaction and Self‐Confidence in Learning Outcomes

The self‐confidence in learning was addressed in questions 6–13; the minimum score was 8, and the maximum was 40. The mean of the Satisfaction with Current Learning scale was 20.20, and the SD was 4.31, while the mean of the Self‐confidence in Learning scale was 31.94, and the SD was 6.49 (Tables 4 and S1).

**TABLE 4:** Results of students' satisfaction and self‐confidence in learning survey.

Continuous variable	Mean	SD	Median	Quartile range	Minimum	Maximum
Satisfaction with current learning	20.20	4.31	20.00	8.00	5.00	25.00
Self‐confidence in learning	31.94	6.49	32.00	8.00	8.00	40.00

Abbreviation: SD, standard deviation.

### 3.6 Correlation of Student Satisfaction, Self‐Confidence in Learning, General Self‐Efficacy and Empathy

The Pearson correlation test showed a correlation between students' satisfaction and self‐confidence in learning and general self‐efficacy (GSE). The findings showed that students' satisfaction and self‐confidence in learning were positively correlated with general self‐efficacy (GSE) (*p* < 0.05). The positive correlation between students' satisfaction and general self‐efficacy (GSE) indicates that self‐efficacy was positively associated with student satisfaction. Also, the positive correlation between self‐confidence in learning and general self‐efficacy (GSE) indicates that self‐efficacy was positively associated with self‐confidence in learning. Also, the findings showed that students' satisfaction, self‐confidence in learning and general self‐efficacy (GSE) were positively correlated with KCES‐R (*p* < 0.05). Specialty and previous simulation experience were not included in the correlation analysis because these variables had no variability in the study sample (Table 5).

**TABLE 5:** Correlation of student satisfaction and self‐confidence in learning, General Self‐Efficacy scale (GSE) and Kiersma–Chen Empathy Scale (KCES) scores.

	Satisfaction	Self‐confidence	GSE	KCES
Satisfaction with current learning	1			
Self‐confidence in learning	0.866*	1		
General Self‐Efficacy Scale (GSE)	0.501*	0.520*	1	
Kiersma–Chen Empathy Scale (KCES‐R)	0.624*	0.632*	0.421*	1

* Correlation is significant at the 0.05 level.

## 4 Discussion

This pre‐test and post‐test quasi‐experimental study aimed to assess the impact of high‐fidelity simulation labs in nursing education on self‐efficacy, empathy and self‐satisfaction/self‐confidence in learning among paediatric nursing students. A significant increase in self‐efficacy scores among nursing students was found following high‐fidelity simulation training, which aligns with existing literature that emphasizes the effectiveness of simulation as a pedagogical tool in nursing education. High‐fidelity simulations provide a realistic and immersive learning environment, allowing students to practice clinical skills and decision‐making in a safe setting. This experiential learning approach has been shown to enhance self‐confidence and satisfaction, which are critical for nursing practice (Toqan et al. 2023). The overall composite score for the General Self‐Efficacy Scale (GSE) showed significant improvement, corroborating previous research indicating that simulation experiences can lead to enhanced self‐efficacy among nursing students (Doğan and Şendir 2022). For instance, a study by Seo and Eom (2021) demonstrated that simulation training significantly improved self‐efficacy and problem solving and clinical competency satisfaction among Korean nursing students, reinforcing that simulation is a valuable educational strategy (Aronson et al. 2022; Seo and Eom 2021). Similarly, Gu and Sok (2020) demonstrated that integrating simulation with innovative instructional models, such as flipped learning, yields comparable improvements, significantly increasing nursing students' self‐efficacy relative to conventional teaching methods (Gu and Sok 2020).

In a different cultural context, a meta‐analysis study by Lei et al. (2022) highlighted that high‐fidelity simulation not only improved knowledge acquisition among nursing students but also enhanced their critical thinking and communication skills. This indicates that while the core benefits of high‐fidelity simulation are consistent globally, cultural factors may influence how these benefits manifest in different educational systems (SAS Institute Inc. 2023; Lei et al. 2022).

The findings regarding empathy suggest a complex relationship between simulation training and empathetic skills, as the overall KCES‐R score did not change significantly following the intervention. While some studies have reported that simulation can foster empathy by allowing students to engage with patient scenarios and reflect on their experiences (Cho and Kim 2024a), the overall lack of significant change in empathy scores indicates that simulation alone may not be sufficient to enhance this skill. While high‐fidelity simulation can improve specific competencies, the development of empathy may require additional structured interventions, such as guided debriefing sessions that focus specifically on the emotional and interpersonal aspects of patient care (Lei et al. 2022; Cho and Kim 2024a). This aligns with findings that emphasize the importance of reflective practices in developing empathy among nursing students. A systematic review emphasized that while high‐fidelity simulations could enhance cognitive competencies, they must be complemented by structured debriefing sessions to develop empathy effectively (Cho and Kim 2024a; Forneris et al. 2015). Additionally, the lack of significant score improvement may be attributed to the complex nature of empathy as a stable psychological trait, which usually requires long‐term longitudinal exposure rather than a single intervention to shift. Furthermore, during acute clinical scenarios; such as anaphylaxis or respiratory distress, students' cognitive focus is heavily directed toward technical safety and protocol management, leaving minimal bandwidth for affective processing.

The positive correlation found among students' satisfaction, self‐confidence in learning and general self‐efficacy is consistent with the literature that highlights the interconnectedness of these constructs in educational settings. Higher self‐efficacy has been linked to greater satisfaction with the learning experience, as students who feel more capable are likely to engage more fully in their education. This relationship underscores the importance of fostering self‐efficacy through effective teaching strategies, such as high‐fidelity simulations, which can lead to improved educational outcomes and student satisfaction. A study by Oanh et al. (2021) supports this by showing that high‐fidelity simulation significantly enhances learner satisfaction and self‐confidence among nursing students (Oanh et al. 2021). Furthermore, a comparative study found similar results, demonstrating that students who engaged in high‐fidelity simulations reported higher satisfaction levels compared to those who underwent traditional learning methods (Forneris et al. 2015; Jawabreh et al. 2025).

### 4.1 Limitations

Although the present study shows important findings, it has some limitations. One limitation is that the present study used a pre‐ and post‐quasi‐experimental design that lacks a control group and random assignment, meaning that observed score changes cannot be definitively isolated from external factors or maturation outside the intervention. Also, the study was performed in one university in Saudi Arabia. Therefore, these data may have limited generalizability due to geographic limitations. Additionally, reliance on self‐reported questionnaires introduces the potential for response bias, as students may have answered in a way they believed was socially desirable rather than reflecting their true internal state. A potential limitation is that participants had prior exposure to general simulation methodologies in earlier courses. While this familiarity may have helped minimize baseline anxiety with simulation equipment, it did not provide specific paediatric knowledge. Future studies could compare paediatric self‐efficacy across cohorts with varying levels of prior simulation experience. Finally, student satisfaction and self‐confidence were evaluated post‐simulation to capture feedback on the specific paediatric scenario and debriefing. Future studies could incorporate longitudinal designs to track confidence changes over time across different clinical courses.

Nevertheless, the present study provides valuable information pertaining to paediatric nursing students' practice in simulation, particularly their self‐efficacy, empathy and satisfaction.

## 5 Conclusions

This pre‐test and post‐test quasi‐experimental study involving undergraduate nursing students in a paediatric nursing course demonstrated that high‐fidelity simulation labs significantly enhance general self‐efficacy. Overall empathy scores did not change significantly following the intervention. Students reported moderate satisfaction and self‐confidence in their learning outcomes, with mean scores reflecting positive experiences. A noteworthy positive correlation among students' satisfaction, self‐confidence in learning and general self‐efficacy suggests that enhancing self‐efficacy can positively influence learning experiences. These findings underscore the critical role of high‐fidelity simulation in fostering self‐efficacy among paediatric nursing students. Nevertheless, further research is needed to better understand the impact of simulation on empathy and to develop comprehensive strategies that address both self‐efficacy and empathetic skills in nursing education, highlighting the importance of a holistic approach to nursing training. Future research could also focus on several key areas, including the long‐term impact of simulation‐based learning on empathy and clinical competencies and the effectiveness of combining simulation with other pedagogical methods like problem‐based or team‐based learning.

## 6 Implications for Nursing Practice

Self‐efficacy, satisfaction and empathy are interrelated factors that significantly impact paediatric nursing students' professional readiness. Improving these attributes through simulation suggests that this pedagogical approach may be a potentially supportive factor for academic performance, better patient care, and a more competent nursing workforce.

The findings of this study have significant implications for nursing education. Given the positive impact of high‐fidelity simulations on self‐efficacy, nursing programmes should consider integrating more simulation‐based learning experiences into their curricula. Specific recommendations include designing simulations that reflect real‐world paediatric scenarios, ensuring that students engage in high‐fidelity and medium‐fidelity simulations to build diverse skill sets, and incorporating interprofessional simulations to foster collaboration with other healthcare disciplines.

However, it is also crucial to address the nuances of empathy development. Nursing programmes could enhance empathy training through structured communication workshops that focus on active listening, empathy‐building exercises and role‐playing scenarios. Additionally, guided reflective activities, such as journaling or group discussions, can help students process their experiences and develop emotional intelligence. These activities should be woven into the simulation curriculum to reinforce the connection between clinical practice and empathetic patient care.

Providing faculty development programmes focused on effective debriefing techniques and emotional support during simulations can also contribute to better learning outcomes for students. By implementing these strategies, nursing education can better prepare students for the complexities of paediatric care while fostering essential empathetic skills.

## Author Contributions

**Yasmine Alabbasi:** conceptualization, funding acquisition, writing – original draft, writing – review and editing, methodology, validation, supervision, formal analysis. **Hibah Bahri:** writing – original draft, writing – review and editing, validation, formal analysis. **Ahlam Almutairi:** conceptualization, investigation, writing – original draft, writing – review and editing, methodology. **Raghad Alqahtani:** conceptualization, investigation, writing – original draft, writing – review and editing, methodology. **Fatima Alsufayan:** conceptualization, investigation, writing – original draft, methodology, writing – review and editing. **Ghaida Alturaysi:** conceptualization, investigation, writing – original draft, writing – review and editing, methodology. **Renad Alshalawi:** conceptualization, investigation, writing – original draft, writing – review and editing, methodology. **Lamya Atin:** conceptualization, investigation, writing – original draft, writing – review and editing, methodology. **Renad Aljuhani:** conceptualization, investigation, writing – original draft, writing – review and editing, methodology. **Savvato Karavasileiadou:** conceptualization, writing – original draft, writing – review and editing, validation, methodology, formal analysis, supervision, data curation.

## Funding

This research was funded by Princess Nourah bint Abdulrahman University, Researchers Supporting Project number (PNURSP2026R390), Princess Nourah bint Abdulrahman University, Riyadh, Saudi Arabia.

## Ethics Statement

This study was conducted in accordance with the Declaration of Helsinki and approved by the Institutional Review Board of Princess Nourah bint Abdulrahman University (PNU) (IRB Log Number: 24–0660).

## Consent

Informed consent was obtained from all subjects involved in this study.

## Conflicts of Interest

The authors declare no conflicts of interest.

## Supporting information


**Table S1:** The percentage of Students' Satisfaction and Self‐Confidence in Learning Questionnaire.

## Data Availability

The data that support the findings of this study are available from the corresponding author upon reasonable request.

## References

[nop270993-bib-0001] Albaqawi, H. , M. Alamri , R. Al‐Dossary , et al. 2025. “Exploring the Impact of Self‐Efficacy Social Support and Learning Environment on Clinical Performance Anxiety in Student Nurses.” Scientific Reports 15, no. 1: 8663. 10.1038/s41598-025-93400-y PMC11906794 40082643

[nop270993-bib-0002] Almotairy, M. M. , M. Algabbashi , S. Alshutwi , et al. 2023. “Nursing Faculty Perceptions of Simulation Culture Readiness in Saudi Universities: A Cross‐Sectional Study.” BMC Nursing 22: 105. 10.1186/s12912-023-01278-w. PMC10079484 37029375

[nop270993-bib-0003] Aronson, B. D. , A. M. H. Chen , M. L. Blakely , M. E. Kiersma , and E. Wicker . 2022. “Evaluation and Revision of the Kiersma–Chen Empathy Scale.” American Journal of Pharmaceutical Education 86, no. 5: 8685. 10.5688/ajpe8685. PMC10159484 34507957

[nop270993-bib-0004] Atta, M. H. R. , H. A. H. Hammad , and N. W. Elzohairy . 2024. “The Role of Empathy in the Relationship Between Emotional Support and Caring Behavior Towards Patients Among Intern Nursing Students.” BMC Nursing 23: 443. 10.1186/s12912-024-02074-w. PMC11212155 38943109

[nop270993-bib-0005] Ayed, A. , M. Z. Malak , R. Al‐Amer , A. Batran , and B. Salameh . 2021. “Effect of High Fidelity Simulation on Perceptions of Self‐Awareness, Empathy, and Patient‐Centered Care Among University Pediatric Nursing Classes.” Clinical Simulation in Nursing 56: 84–90. 10.1016/j.ecns.2021.04.005.

[nop270993-bib-0007] Cho, M. K. , and M. Y. Kim . 2024b. “Effectiveness of Simulation‐Based Interventions on Empathy Enhancement Among Nursing Students: A Systematic Literature Review and Meta‐Analysis.” BMC Nursing 23: 319. 10.1186/s12912-024-01944-7. PMC11088026 38734606

[nop270993-bib-0006] Cho, M. , and M. Y. Kim . 2024a. “Effectiveness of Simulation‐Based Interventions on Empathy Enhancement Among Nursing Students: A Systematic Literature Review and Meta‐Analysis.” BMC Nursing 23: 319. 10.1186/s12912-024-01944-7. PMC11088026 38734606

[nop270993-bib-0008] Deligianni, A. , M. Kyriakidou , E. Kaba , et al. 2017. “Empathy Equals: The Meaning of Empathy as It Perceived by Greek Nurse Students—A Qualitative Study.” Global Journal of Health Science 9: 171–180. 10.5539/gjhs.v9n1p171.

[nop270993-bib-0009] Doğan, P. , and M. Şendir . 2022. “Effect of Different Simulation Methods in Nursing Education on Critical Thinking Dispositions and Self‐Efficacy Levels of Students.” Thinking Skills and Creativity 1, no. 45: 101112.

[nop270993-bib-0010] Eltaib, F. A. , F. J. Alanazi , F. A. Mersal , and T. H. Aboelola . 2024. “Perceptions Regarding Attitude Towards Simulation‐Based Learning Among Nursing Students in Saudi Arabia: A Cross‐Sectional Study.” Belitung Nursing Journal 10: 578–584. 10.33546/bnj.3438. PMC11474263 39416354

[nop270993-bib-0011] Forneris, S. G. , C. Peden‐McAlpine , and D. Tschannen . 2015. “The Impact of High‐Fidelity Simulation on Nursing Students' Cognitive Competencies and Empathy Development: A Systematic Review.” Nurse Education Today 35: 641–646. 10.1016/j.nedt.2015.01.001.

[nop270993-bib-0012] Franklin, A. E. , P. Burns , and C. S. Lee . 2014. “Psychometric Testing on the NLN Student Satisfaction and Self‐Confidence in Learning, Simulation Design Scale, and Educational Practices Questionnaire Using a Sample of Pre‐Licensure Novice Nurses.” Nurse Education Today 34: 1298–1304. 10.1016/j.nedt.2014.06.011. 25066650

[nop270993-bib-0013] Gordon, J. A. , W. M. Wilkerson , D. W. Shaffer , and E. G. Armstrong . 2001. ““Practicing” Medicine Without Risk: Students' and Educators' Responses to High‐Fidelity Patient Simulation.” Academic Medicine 76, no. 5: 469–472. 10.1097/00001888-200105000-00019 11346525

[nop270993-bib-0014] Gu, M. , and S. Sok . 2020. “Effects of Simulation Practicum Using Flipped Learning for Korean Nursing Students.” International Journal of Environmental Research and Public Health 17: 6829. 10.3390/ijerph17186829. PMC7559239 32962057

[nop270993-bib-0015] Guerrero, J. G. , D. M. Attallah , N. H. Gomma , and S. A. Ali . 2024. “Improvements in Practising Nurses' Knowledge, Skills, Self‐Efficacy, Confidence, and Satisfaction After a Simulated Clinical Experience of Caring for a Patient Undergoing Chemotherapy: A Quasi‐Experimental Study.” BMC Nursing 23, no. 1: 66. 10.1186/s12912-024-01727-0. PMC10807190 38267925

[nop270993-bib-0016] Hodrob, A. M. S. , M. Z. Malak , and A. Ayed . 2024. “Effect of High‐Fidelity Simulation Airway Management Training Program on Nursing Students' Performance, Satisfaction, and Self‐Confidence in Palestine.” Interactive Learning Environments 32, no. 1: 325–339. 10.1080/10494820.2022.2086576.

[nop270993-bib-0017] Jacobs, A. , and I. Venter . 2017. “Standardised Patient‐Simulated Practice Learning: A Rich Pedagogical Environment for Psychiatric Nursing Education.” African Journal of Health Professions Education 9: 107. 10.7196/ajhpe.2017.v9i3.806.

[nop270993-bib-0018] Jawabreh, N. , A. Hamdan‐Mansour , L. Harazne , and A. Ayed . 2025. “Effectiveness of High‐Fidelity Simulation on Practice, Satisfaction, and Self‐Confidence Among Nursing Students in Mental Health Nursing Class.” BMC Nursing 24, no. 1: 622. 10.1186/s12912-025-03300-9. PMC12125790 40450268

[nop270993-bib-0035] Jeffries, P. R. , and M. A. Rizzolo . 2006. “Designing and Implementing Models for the Innovative Use of Simulation to Teach Nursing Care of ill Adults and Children: A National Multi‐Site, Multi‐Method Study.” National League for Nursing: 500–505.

[nop270993-bib-0019] Koukourikos, K. , A. Tsaloglidou , L. Kourkouta , et al. 2021. “Simulation in Clinical Nursing Education.” Acta Informatica Medica 29, no. 1: 15. 10.5455/aim.2021.29.15-20. PMC8116070 34012208

[nop270993-bib-0020] Lei, Y.‐Y. , L. Zhu , Y. T. R. Sa , and X.‐S. Cui . 2022. “Effects of High‐Fidelity Simulation Teaching on Nursing Students' Knowledge, Professional Skills and Clinical Ability: A Meta‐Analysis and Systematic Review.” Nurse Education in Practice 60: 103306. 10.1016/j.nepr.2022.103306. 35202957

[nop270993-bib-0021] Lewis, L. S. , L. M. Rebeschi , and E. Hunt . 2022. “Nursing Education Practice Update 2022: Competency‐Based Education in Nursing.” SAGE Open Nursing 8: 23779608221140774. 10.1177/23779608221140774. PMC9685206 36437897

[nop270993-bib-0022] Mohamed, S. , and I. Fashafsheh . 2019. “The Effect of Simulation‐Based Training on Nursing Students' Communication Skill, Self‐Efficacy and Clinical Competence for Nursing Practice.” Open Journal of Nursing 9: 855–869. 10.4236/ojn.2019.98064.

[nop270993-bib-0023] Moudatsou, M. , A. Stavropoulou , A. Philalithis , and S. Koukouli . 2020. “The Role of Empathy in Health and Social Care Professionals.” Healthcare 8: 26. 10.3390/healthcare8010026. PMC7151200 32019104

[nop270993-bib-0024] Mujamammi, A. H. , S. A. Alqahtani , F. A. Alqaidi , et al. 2024. “Evaluation of Medical Students' Satisfaction With Using a Simulation‐Based Learning Program as a Method for Clinical Teaching.” Cureus 16, no. 4: e59364. 10.7759/cureus.59364. PMC11137646 38817460

[nop270993-bib-0025] Nguyen, P. V. , H. T. Pham , and V. H. Dang . 2026. “Self‐Efficacy, Engagement, Learning Outcomes, Well‐Being, and Overall Perceived Satisfaction in University Students.” European Journal of Psychology of Education 41, no. 2: 82. 10.1007/s10212-026-01147-5.

[nop270993-bib-0026] Nguyen, W. , I. Fromer , M. Remskar , and E. Zupfer . 2023. “Development and Implementation of Video‐Recorded Simulation Scenarios to Facilitate Case‐Based Learning Discussions for Medical Students' Virtual Anesthesiology Clerkship.” MedEdPORTAL 19: 11306. 10.15766/mep_2374-8265.11306 PMC10070881 37025196

[nop270993-bib-0027] Oanh, T. T. H. , N. T. Y. Hoai , and P. T. Thuy . 2021. “The Relationships of Nursing Students' Satisfaction and Self‐Confidence After a Simulation‐Based Course With Their Self‐Confidence While Practicing on Real Patients in Vietnam.” Journal of Educational Evaluation for the Health Professions 18: 16. 10.3352/jeehp.2021.18.16. PMC8382883 34325495

[nop270993-bib-0028] Oermann, M. H. , and K. B. Gaberson . 2021. Evaluation and Testing in Nursing Education. Springer Publishing Company.

[nop270993-bib-0029] Salameh, A. K. , M. Z. Malak , F. A. El‐Qirem , M. Alhussami , and M. El‐hneiti . 2024. “Effect of Virtual Reality Simulation as a Teaching Strategy on Nursing Students' Satisfaction, Self‐Confidence, Performance, and Physiological Measures in Jordan.” Teaching and Learning in Nursing 19, no. 1: e235–e241.

[nop270993-bib-0030] SAS Institute Inc . 2023. SAS/STAT 15.3 User's Guide. SAS Institute Inc.

[nop270993-bib-0031] Schwarzer, R. , and M. Jerusalém . 1995. “Generalized Self‐eEfficacy Scale.” In Measures in Health Psychology: A User's Portfolio. Casual and Control Beliefs. NFER‐NELSON.

[nop270993-bib-0032] Seo, Y. H. , and M. R. Eom . 2021. “The Effect of Simulation Nursing Education Using the Outcome‐Present State‐Test Model on Clinical Reasoning, the Problem‐Solving Process, Self‐Efficacy, and Clinical Competency in Korean Nursing Students.” Healthcare 9, no. 3: 243. 10.3390/healthcare9030243. PMC7996191 33668362

[nop270993-bib-0033] Toqan, D. , A. Ayed , I. A. Khalaf , and M. Alsadi . 2023. “Effect of High‐Fidelity Simulation on Self‐Satisfaction and Self‐Confidence Among Nursing Students.” SAGE Open Nursing 9: 23779608231194403. 10.1177/23779608231194403. PMC10424544 37584033

[nop270993-bib-0034] Van Dijke, J. , I. van Nistelrooij , P. Bos , and J. Duyndam . 2020. “Towards a Relational Conceptualization of Empathy.” Nursing Philosophy 21, no. 3: e12297. 10.1111/nup.12297. PMC9286577 32077225

