# Supplementary material for: Effect of High‐Fidelity Simulation on Self‐Efficacy, Empathy and Satisfaction Among Undergraduate Paediatric Nursing Students: A Quasi‐Experimental Study
Source: Nurs Open. 2026 Nov 13;13(11):e70993. doi: 10.1002/nop2.70993 (PMC13768585; doi:10.1002/nop2.70993)
Supplement: Supplementary file 1 [file NOP2-13-e70993-s001.docx]

**Table S1.** The Percentage of Students’ Satisfaction and Self-Confidence in Learning Questionnaire.

| Questions: | Frequency | (%) |
| --- | --- | --- |
| **Satisfaction with Current Learning** |  |  |
| 1. The teaching methods used in this simulation were helpful and effective. |  |  |
| Strongly Disagree | 3 | 2.10 |
| Disagree | 8 | 5.59 |
| Undecided | 26 | 18.18 |
| Agree | 51 | 35.66 |
| Strongly Agree | 55 | 38.46 |
| 2. The simulation provided me with a variety of learning materials and activities to promote my learning the Pediatric medical surgical curriculum |  |  |
| Strongly Disagree | 4 | 2.80 |
| Disagree | 5 | 3.50 |
| Undecided | 25 | 17.48 |
| Agree | 56 | 39.16 |
| Strongly Agree | 53 | 37.06 |
| 3. I enjoyed how my instructor taught the simulation |  |  |
| Strongly Disagree | 3 | 2.10 |
| Disagree | 4 | 2.80 |
| Undecided | 25 | 17.48 |
| Agree | 58 | 40.56 |
| Strongly Agree | 53 | 37.06 |
| 4- The teaching materials used in this simulation were motivating and helped me to learn |  |  |
| Strongly Disagree | 4 | 2.80 |
| Disagree | 5 | 3.50 |
| Undecided | 24 | 16.78 |
| Agree | 61 | 42.66 |
| Strongly Agree | 49 | 34.27 |
| 5- The way my instructor(s) taught the simulation was suitable to the way I learn. |  |  |
| Strongly Disagree | 3 | 2.10 |
| Disagree | 5 | 3.50 |
| Undecided | 29 | 20.28 |
| Agree | 53 | 37.06 |
| Strongly Agree | 53 | 37.06 |
| **Self-confidence in Learning** |  |  |
| 6- I am confident that I am mastering the content of the simulation activity that my instructors presented to me. |  |  |
| Strongly Disagree | 5 | 3.50 |
| Disagree | 4 | 2.80 |
| Undecided | 25 | 17.48 |
| Agree | 63 | 44.06 |
| Strongly Agree | 46 | 32.17 |
| 7- I am confident that this simulation covered critical content necessary for the mastery of Pediatric medical surgical curriculum. |  |  |
| Strongly Disagree | 4 | 2.80 |
| Disagree | 5 | 3.50 |
| Undecided | 24 | 16.78 |
| Agree | 60 | 41.96 |
| Strongly Agree | 50 | 34.97 |
| 8- I am confident that I am developing the skills and obtaining the required knowledge from this simulation to perform necessary tasks in a clinical setting |  |  |
| Strongly Disagree | 4 | 2.80 |
| Disagree | 4 | 2.80 |
| Undecided | 25 | 17.48 |
| Agree | 59 | 41.26 |
| Strongly Agree | 51 | 35.66 |
| 9- My instructors used helpful resources to teach the simulation |  |  |
| Strongly Disagree | 4 | 2.80 |
| Disagree | 5 | 3.50 |
| Undecided | 29 | 20.28 |
| Agree | 54 | 37.76 |
| Strongly Agree | 51 | 35.66 |
| 10- It is my responsibility as the student to learn what I need to know from this simulation activity |  |  |
| Strongly Disagree | 4 | 2.80 |
| Disagree | 9 | 6.29 |
| Undecided | 22 | 15.38 |
| Agree | 57 | 39.86 |
| Strongly Agree | 51 | 35.66 |
| 11- I know how to get help when I do not understand the concepts covered in the simulation. |  |  |
| Strongly Disagree | 9 | 6.29 |
| Disagree | 4 | 2.80 |
| Undecided | 28 | 19.58 |
| Agree | 57 | 39.86 |
| Strongly Agree | 45 | 31.47 |
| 12- I know how to use simulation activities to learn critical aspects of these skills |  |  |
| Strongly Disagree | 4 | 2.80 |
| Disagree | 6 | 4.20 |
| Undecided | 25 | 17.48 |
| Agree | 58 | 40.56 |
| Strongly Agree | 50 | 34.97 |
| 13- It is the instructor's responsibility to tell me what I need to learn of the simulation activity content during class time. |  |  |
| Strongly Disagree | 6 | 4.20 |
| Disagree | 7 | 4.90 |
| Undecided | 20 | 13.99 |
| Agree | 57 | 39.86 |
| Strongly Agree | 53 | 37.06 |
| Total | 143 | 100 |

The National League for Nursing (Jeffries and Rizzolo, 2006). Adapted version of the Student Satisfaction and Self-Confidence in Learning Scale was used. Items 2 and 7 were adapted to change the focus from a medical-surgical curriculum to a pediatric medical-surgical.
